# Accuracy of digital planning in zygomatic implants

**DOI:** 10.1186/s40729-021-00350-x

**Published:** 2021-07-22

**Authors:** B. Xing Gao, O. Iglesias-Velázquez, F. G.F. Tresguerres, A. Rodríguez González Cortes, I. F. Tresguerres, R. Ortega Aranegui, R. M. López-Pintor, J. López-Quiles, J. Torres

**Affiliations:** 1 Department of Dental Clinical Specialties, Faculty of Dentistry, Complutense University, Plaza Ramón y Cajal, s/n (Ciudad Universitaria), 28040 Madrid, Spain; 2 Department of Oral and Maxillofacial Surgery, Faculty of Dentistry, University of Sao Paulo, Sao Paulo, Brazil

**Keywords:** Zygomatic implants, Digital planning, Surgical guides

## Abstract

**Background:**

Zygomatic implants have been described as a therapeutic alternative for patients with severe maxillary atrophy in order to avoid bone augmentation procedures. Taking that into account, in these treatments, the key factor is the position of the implant, the virtual surgical planning (VSP) is widespread among most clinicians before surgery on the patient. However, there are no studies which evaluate the clinical relevance of these VSP.

The aim of this study is to determine whether digital planning on zygomatic implants has any influence on the implant dimensions and position, even when performing conventional surgery afterwards.

**Results:**

Fourteen zygomatic implants were placed in four patients. Pre-operative and post-operative helicoidal computed tomography were performed to each patient to allow the comparison between the digital planning and the final position of implants. Tridimensional deviation (TD), mesio-distal deviation (MDD), bucco-palatine deviation (BPD), and apico-coronal deviation (ACD) were evaluated as well as angular deviation (AD). Significative differences in apical TD were observed with a mean of 6.114 ± 4.28 mm (*p* < 0.05). Regarding implant position, only implants placed in the area of the first right molar reported significant differences (*p* < 0.05) for ACD. Also, implant length larger than 45 mm showed BPD significative differences (*p* < 0.05).

**Conclusions:**

Zygomatic implant surgery is a complex surgical procedure, and although VSP is a useful tool which helps the clinician determine the number and the length of zygomatic implants as well as its proper position, surgical experience is still mandatory.

## Background

Full-arch implant rehabilitations are currently one of the most common and predictable treatments in daily clinical practise, reaching high long-term survival rates [1]. As widely known, tooth loss leads to generalized resorption of alveolar process which can produce severe resorptions at maxillae and mandibular bone, preventing implant treatments due to the lack of sufficient bone height and width [2, 3]. Furthermore, the absence of dental stimuli and the positive pressure of the maxillary sinus increase, generating a progressive pneumatization of this cavity, further compromising bone availability [4, 5]. Several techniques have been developed with the aim of achieving sufficient bone augmentation to allow dental implant placement. These procedures, however, usually require a long surgical time, multiple interventions, and are oftenly associated with severe complications and high morbidity [6].

In 1989, Brånemark et al. [6] described the use of zygomatic implants as a therapeutic alternative for patients with severe maxillary atrophy to avoid bone augmentation procedures. This technique consisted in placing a long dental implant through maxillary sinus, to be anchored in zygomatic bone. Nevertheless, to avoid functional and aesthetic complications associated with pronounced concavities in the lateral maxillary wall [7], as well as other complications such as sinusitis [8, 9], Stella y Warner et al. [10] developed the “sinus slot” technique.

One of the key factors for zygomatic implants is the implant positioning. It is essential that the apex of the implant is placed in the areas of zygomatic bone with higher bone density and reaching the greater bone to implant contact (BIC) in all its course as far as possible. Furthermore, there is a wide variety of zygomatic implant brands. In this study, the implants used in all cases were NobelZygoma®. To ensure the correct osseointegration of these implants and knowing that they have an apical diameter of 3.75 mm, the minimum amount of bone required is 5.75 mm. Takamaru et al. [11] carried on a study in which determined that the most suitable areas for implant placing were the upper posterior area and the central area of zygomatic bone. On the other hand, authors such as Hung et al. [12] have determined, by using CBCT studies, several more suitable areas for the implant apex positioning, being the upper posterior area (A3) and the central area (B1) of the zygomatic bone the most favourable, agreeing with the results of Takamaru et al. [11]. Currently, one approach of zygomatic implant treatments is based in the “Quad Zygoma” concept which seeks to achieve complete upper restorations of atrophic maxilla by placing four implants [13, 14].

Scientific advances in computerized radiology machines [15] have allowed improvements in diagnostic and therapeutic tools. Although zygomatic implants surgical guides have been demonstrated to be inaccurate, most clinicians usually perform digital planification and virtual surgery prior to the intervention on the patient [16].

Nevertheless, and to the best of our knowledge, in current scientific literature, there are no studies which evaluate the clinical relevance of these VSP or studies which compares the variations in the position and angulation of implants placed conventionally with the previous digital planning.

The aim of this study is to evaluate if there are any advantages in using three-dimensional digital planning software for zygomatic implants placed in a conventional manner.

## Material and method

### Patients

We included in our study patients without gender and age restriction, those patients in which the use of zygomatic implants was indicated because of the occurrence of extreme bone maxillary atrophy that prevents conventional implant placement. Moreover, patients with severe systemic diseases classified as ASA III or IV (American Society of Anesthesiology) or smokers of more than 10 cigarettes per day were excluded. Additionally, all participants understood the objectives of the study explained and were given an informed written consent which was signed before performing any procedure. Finally, four patients who had 14 zygomatic implants placed, fulfilled the inclusion criteria, and were enrolled in our study. All four patients completed both pre-operative and post-operative radiographic explorations; therefore, no patient was excluded from the study. The study was conducted in accordance with the principles outlined in the Declaration of Helsinki 1964 on clinical studies involving humans (Table 1).

**Table 1:** Sample characteristics

Patient	Implant position	Implant type	Planned implant length (mm)	Final implant length (mm)	Implant diameter (mm)	Implant apical diameter (mm)
1	16	Nobel	35	35	4.1	3.75
	14	Nobel	40	40	4.1	3.75
	24	Nobel	40	40	4.1	3.75
	26	Nobel	35	35	4.1	3.75
2	16	Nobel	40	40	4.1	3.75
	14	Sweden	4 × 8.5	4 × 8.5	NA	NA
	24	Sweden	4 × 8.5	4 × 8.5	NA	NA
	26	Nobel	40	40	4.1	3.75
3	16	Nobel	50	50	4.1	3.75
	14	Nobel	52.5	52.5	4.1	3.75
	24	Nobel	52.5	52.5	4.1	3.75
	26	Nobel	50	50	4.1	3.75
4	16	Nobel	45	47.5	4.1	3.75
	14	Nobel	47.5	47.5	4.1	3.75
	24	Nobel	47.5	47.5	4.1	3.75
	26	Nobel	45	47.5	4.1	3.75

### Digital pre-operative planning

**Fig. 1:**
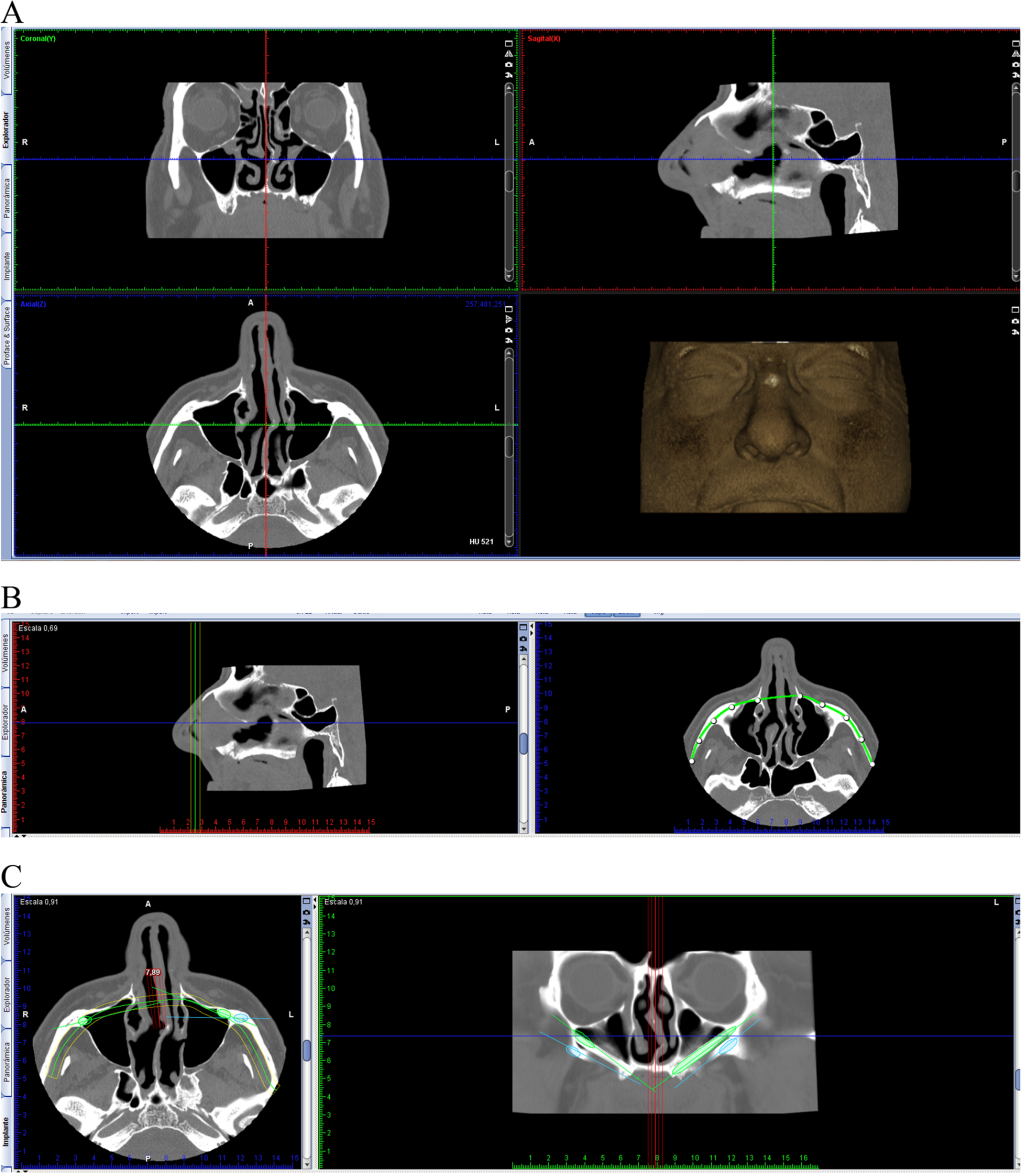
Case plannification

All four patients were assessed clinically, and a pre-operative helicoidal computed tomography was performed into Dr. Ortega buccofacial diagnosis clinic (Madrid, Spain) by using helicoidal CT Optima CT 520, General Electric (General Electric Medical Systems, Milwaukee). Radiologic records data was exported in DICOM 3 (Digital Imaging and Communication On Medicine) format file to Planmeca Romexis® 3D software, which was used for every case planning by the same surgeon who performed the live surgery (Fig. 1a). Axial axis was displaced until zygomatic arch was completely visible, and as marked using the software tools (Fig. 1b). NobelZygoma® implants were chosen from the implant library of the software and were used to perform the virtual surgical planning over the radiological data (Fig. 1c). Finally, a single DICOM format file, containing the unified data from the radiological records and the digital planning, was generated automatically by Planmeca Romexis® software.

### Surgical procedure

All four patients were operated by the same surgeon which performed by using 4% articaine and 1:100000 epinephrine as local anesthetic agent (Ultracain DS forte, Sanofi-Aventis, Germany). We designed a horizontal crestal incision in the area of upper maxilla in which zygomatic implants were going to be placed and a full-thickness mucoperiosteal flap was raised to the infraorbitary foramen in order to visualize hard tissue. Implant drilling sequence was performed, and implants were placed following the previous digital planification. Finally, the healing abutment was placed and after repositioning of the flaps, the wound was closed with 4–0 nylon sutures (Supramid, Serag-Wiessner, Germany). Cuadro de los pacientes con las posiciones de los implantes.

### Data analysis

**Fig. 2:**
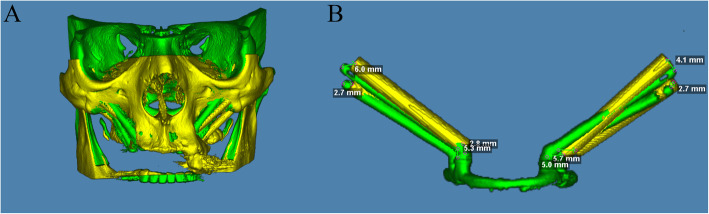
DICOM files overlayering and zygomatic implants isolation

Three months after surgery, a post-operative CT was performed, obtaining a second DICOM format file. Both, DICOM format files were exported to Dolphin Imaging 11.95 Premium® and were overlayered by using three reference points: right inferior orbital rim (P1), anterior nasal spine (P2), and left inferior orbital rim (P3) (Fig. 2a). Bone tissues were removed, and zygomatic implants were isolated to perform the measurements (Fig. 2b). Tridimensional deviation (TD), mesio-distal deviation (MDD), bucco-palatine deviation (BPD), and apico-coronal deviation (ACD) were evaluated at the level of the apex and the neck of the implant. Moreover, angular deviation (AD) and implant length (IL) were also measured.

### Statistical analysis

The Shapiro-Wilk normality test with a confidence interval of 95% yielded that IL, apical MDD, neck TD, neck MDD, and AD conformed to normal distribution, although the rest of the variables did not adjust to it. Due to the small sample size, parametric and not parametric test were carried on by using SPSS software. We carried on the following:

- *T* test and Wilcoxon signed-rank test for a single sample to all dependent variables for assess its clinical relevance.
- Kruskal-Wallis test and ANOVA test for more than two independent samples according to the position of the implant.
- Mann-Whitney *U* test and *T* test for two independent samples according to implant length.

## Results

Fourteen zygomatic implants were placed in 4 patients, 2 males, and 2 females with a mean age of 48.75 years old. Preoperative VSP took a mean time of 10 min and 20 s. Twelve of the 14 placed implants had the same length as digitally planed (85.72%) (Table 1).

Regarding implant deviations, apical TD was the highest registered value with a mean of 6.114 ± 4.28 mm. One implant in position 1.6 had a greater apical TD deviation, (more than 10 mm). This result is due to limited buccal aperture of the patient, a variable difficult to take into account in VSP. While cervical ACD was the lower levels, with a mean deviation of 1.993 ± 1.83 mm. The analysis showed grater variations in BPD and MDD at apex and neck levels respectively with mean values of 4.221 ± 4.3 mm and 3.279 ± 2.34 mm. With respect to the AD, the mean value was 8.357 ± 5.3°. The *T* test and the Wilcoxon-test showed significant differences for all dependent variables (*p* < 0.05) (Table 2).

**Table 2:** Mean values of different deviations

Type of deviation	Mean	SD	Significance
Apical TD	6.114	4.28	*p* > 0.05
Apical MDD	2.236	1.29	
Apical BPD	4.221	4.3	
Apical ACD	2.75	2.16	
Cervical TD	4.986	2.66	
Cervical MDD	3.279	2.34	
Cervical BPD	2.879	2.31	
Cervical ACD	1.993	1.83	
AD	8.357	5.3	

### Influence of implant position

**Fig. 3:**
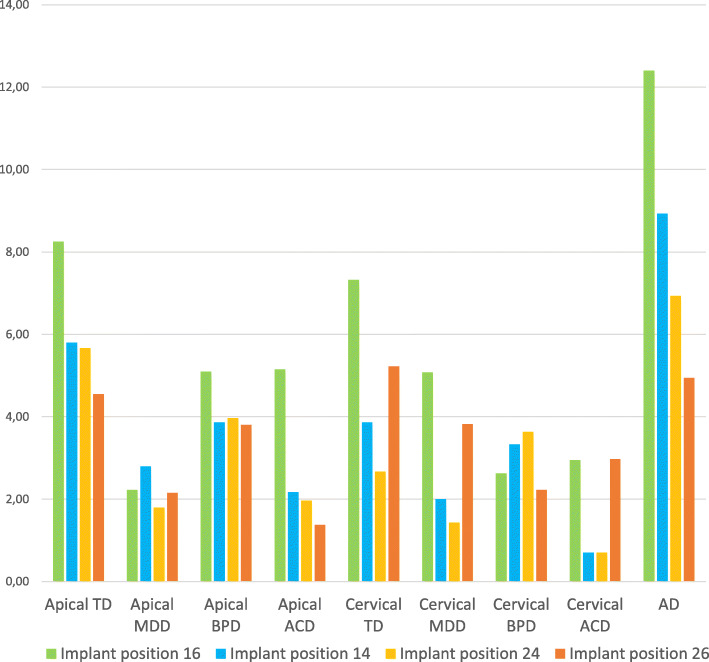
Influence of implant position in deviations

Regarding TD, the zygomatic implants placed in the most posterior areas (first molar or second bicuspid) presented more deviations compared with those placed in more anterior (first bicuspid or canine) areas, being the first quadrant the one that presented greater deviation both at the apical and cervical levels. Apical and cervical MDD values were quite homogeneous, being those implants placed in the lateral zone of the second quadrant which presented less variations. Apical BPD showed higher values than cervical BPD, similar to the TD mentioned previously. Apical ACD yielded higher values at implants placed in the posterior region of the first quadrant, decreasing towards the second quadrant. In the same way, cervical ACD registered higher values in posterior zone. AD resulted in lower values in posterior zone of second quadrant, increasing towards the contralateral posterior zone. The ANOVA test reported significant differences (*p* < 0.05) for the apical ACD with respect to the implant position comparing VSP with the final position of the implants, but no other variable resulted in significant differences (Fig. 3).

### Influence of implant length

**Fig. 4:**
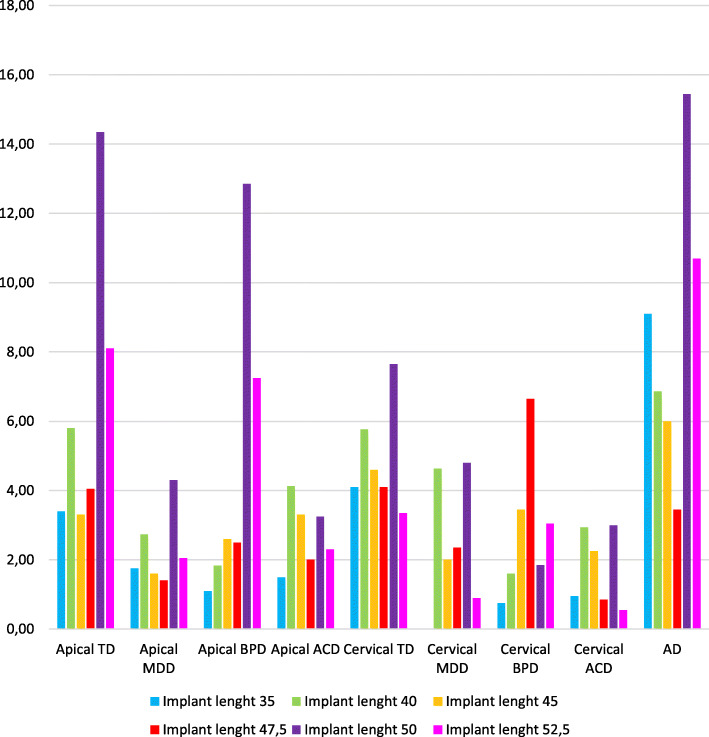
Influence of implant length in deviations

Apical and cervical TD seemed to be higher in posterior zygomatic implants and were correlated with implant size, being higher in 50 mm implants. In the same way, apical MDD showed higher values in 50 mm, 52.5 mm, and 40 mm implants, even more in those placed in posterior zone. Although MDD at the neck level was homogeneous, apical BPD yielded heterogeneous values, reaching less deviation in 35 mm implants (1.1 ± 0.8 mm) compared with 50 mm implants (12.85 ± ,15 mm). MDD at the neck of the implant was higher in 47.5 mm implants. Regarding apical and cervical ACD, values were homogeneous for all implant lengths. Moreover, the higher the length of the implant was, the higher AD was registered. Due to the small sample size, to help perform the statistical analysis, two groups were generated, and the implants were classified according to whether they were larger or smaller than 45 mm. *T* test and Mann-Whitney *U* test showed significant differences (*p* < 0.05) for apical BPD in those implants larger than 45 mm (Fig. 4)

## Discussion

To the best of our knowledge, this is the first study in current scientific literature that evaluates the clinical utility of VSP for the placement of zygomatic implants in a conventional manner. The present study shows higher apical mean deviations compared with cervical deviations regardless implant length or position, being the TD and AD the highest. Furthermore, we observed some differences due to the influence of the length and the position of the implants, which seems to be accentuated when the implants are placed posteriorly and with the increase in the length of the implants.

VSP has been significantly advance in implantology, allowing clinicians to visualize the surgical procedure before performing the intervention over the patient. Moreover, it provides complementary information which helps to determine the number of implants, the adequate implant length, and its proper position [16]. Nevertheless, it requires a variable learning curve depending on individual characteristics.

CAD-CAM technology [17] has helped in developing minimally invasive surgical techniques guided by intraoral splints [18] or computer-assisted procedures [19–21]. Treatments with conventional implants performed under surgical guides have achieved survival rates similar to conventional procedures [22], providing greater accuracy and precision than freehand techniques [23]. On the other hand, due to variations in the position and angulation of implants with respect to the previous computer-assisted planification zygomatic implants, surgical guides have demonstrated to be highly imprecise [18, 24].

With respect to apical variations observed, BPD are higher than MDD and ACD probably due to midface anatomy. After implant placement, the apex is located between the orbit anteriorly and the dermis of the face laterally. These two areas are susceptible to different complications such as orbit invasion, although very rare, has been described by various authors [25, 26], or dermis perforation, which could cause cutaneous fistulas [27]. For these reasons, the posterior area was where we observed most of the variations, because it is the zone that offers more manoeuvring possibilities. Regarding cervical variations, due to the clear clinical visualization of implant neck, higher and smaller deviations were MDD and ACD respectively because of the properly position and depth of the implant at bone crest level determines the result of the prosthetic phase [28] and reduce inflammatory complications, such as mucositis and peri-implantitis, as what occurred in conventional implants [29].

In addition, we observed some differences related to implant position and implant length. It seems that the more posterior the implant position, the more variations are recorded, more specifically in those implants placed in the right first molar position. These variations are probably associated with the length of the drills and mouth opening limitation, reason of which it is strongly recommended to start the drilling sequence in the posterior region [13, 14].

On the other hand, we observed some differences depending on whether the implant was in the first or second quadrant. These variations may be related to the right-handed condition of the operator. Greater deviation in most of the measured parameters in the first quadrant is probably due to the difficulty of handling rotary instruments in a non-comfortable working position.

However, the present study has some limitations. Due to the pilot study condition of our investigation, sample size is quite reduced, preventing us to reach statistical significance in most of compared variables related to implant length and position. Nevertheless, these preliminary data have allowed us to calculate that 35 patients are the necessary sample size to perform a second study.

Taking into account the above data, although the virtual planning of this type of surgery can help to determine certain parameters related to the characteristics of the implant and its position, the absence of an effective method that transfers this virtual planning to the surgical field, causes that the surgery is carried out in a conventional way, causing certain differences between the final implant position and the virtually planned one.

## Conclusion

Zygomatic implant surgery is a complex surgical procedure, and although VSP is a useful tool which helps the clinician determine the number and the length of zygomatic implants as well as its proper position, surgical experience is still mandatory

Although our study reports some differences between VSP and the final treatment, we strongly suggest that further developments are necessary to allow the VSP to be transferred in an accurate manner from the computer to the surgery, by static or navigation-guided surgery.

## Data Availability

The datasets used and/or analyzed during the current study are available from the corresponding author on reasonable request.

## Abbreviations

VSP: Virtual surgical planning
BIC: Bone to implant contact
ASA: American Society of Anaesthesiologists
Helicoidal CT: Helicoidal computed tomography
TD: Tridimensional deviation
MDD: Mesio-distal deviation
BPD: Bucco-palatine deviation
ACD: Apico-coronal deviation
AD: Angular deviation
IL: Implant length

## References

[1] Chappuis V, Buser R, Bragger U, Bornstein MM, Salvi GE, Buser D (2013). Long-term outcomes of dental implants with a titanium plasma-sprayed surface: a 20-year prospective case series study in partially edentulous patients. Clin Implant Dent R.

[2] Chrcanovic BR, Nogueira MH, Abreu G (2013). Survival and complications of zygomatic implants: a systematic review. Oral Maxillofac Surg.

[3] Agliardi EL, Romeo D, Panigatti S, de Araújo Nobre M, Maló P (2017). Immediate full-arch rehabilitation of the severely atrophic maxilla supported by zygomatic implants: a prospective clinical study with minimum follow-up of 6 years. Int J Oral Maxillofac Surg.

[4] Wehrbein H, Diedrich P (1992). Progressive pneumatisation of the basal maxillary sinus after extraction and space closure. Fortschr Kieferorthop.

[5] Lombardi T, Bernardello F, Berton F, Porrelli D, Rapani A, Camurri Piloni A (2018). Efficacy of alveolar ridge preservation after maxillary molar extraction in reducing crestal bone resorption and sinus pneumatization: a multicenter prospective case-control study. Biomed Res Int.

[6] Brånemark PI (1998). Surgery and fixture installation. Zygomaticus fixture clinical procedures (ed 1).

[7] Aparicio C (2011). A proposed classification for zygomatic implant patients based on the zygoma anatomy guided approach (ZAGA): a cross-sectional survey. Eur J Oral Implantol.

[8] Aparicio C, Ouazzani W, Aparicio A, Fortes V, Muela R, Pascual A (2010). Immediate/early loading of zygomatic implants: clinical experiences after 2 to 5 years of follow-up. Clin Implant Dent Relat Res.

[9] Molinero-Mourelle P, Baca-Gonzalez L, Gao B, Saez-Alcaide LM, Helm A, Lopez-Quiles J (2016). Surgical complications in zygomatic implants: a systematic review. Med Oral Patol Oral Cir Bucal.

[10] Stella JP, Warner MR (2000). Sinus slot technique for simplification and improved orientation of zygomaticus dental implants: a technical note. Int J Oral Maxillofac Implants.

[11] Takamaru N, Nagai H, Ohe G, Tamatani T, Sumida K, Kitamura S (2016). Measurement of the zygomatic bone and pilot hole technique for safer insertion of zygomaticus implants. Int J Oral Maxillofac Surg.

[12] Hung K, Ai Q, Fan S, Wang F, Huang W, Wu Y (2017). Measurement of the zygomatic region for the optimal placement of quad zygomatic implants. Clin Implant Dent Relat Res.

[13] Davó R, David L (2019). Quad Zygoma. Oral Maxillofacial Surg Clin North Am.

[14] Aboul-Hosn Centenero S, Lázaro A, Giralt-Hernando M, Hernández-Alfaro F. Zygoma quad compared with 2 zygomatic implants. Implant Dent. 2018;27(2):246–53. 10.1097/ID.0000000000000726 29394179

[15] Lombardo G, D’Agostino A, Trevisiol L, Romanelli MG, Mascellaro A, GomezLira M (2016). Clinical, microbiological and radiological assessment of soft and hard tissues surrounding zygomatic implants: a retrospective study, oral surgery, oral medicine, oral pathology and oral radiology.

[16] Jung RE, Schneider D, Ganeles J, Wismeijer D, Zwahlen M, Hämmerle CH (2009). Computer technology applications in surgical implant dentistry: a systematic review. Int J Oral Maxillofac Implants.

[17] Verstreken K, Van Cleynenbreugel J, Marchal G, Naert I, Suetens P, van Steenberghe D (1996). Computer-assisted planning of oral implant surgery: a three-dimensional approach. Int J Oral Maxillofac Implants.

[18] Chrcanovic BR, Oliveira DR, Custódio AL (2010). Accuracy evaluation of computed tomography-derived stereolithographic surgical guides in zygomatic implant placement in human cadavers. J Oral Implantol.

[19] Schramm A, Gellrich NC, Schimming R, Schmelzeisen R (2000). Rechnergestützte insertion von Zygomatikumimplantaten (Brånemark-system) nach ablativer Tumorchirurgie. Mund Kiefer Gesichtschir.

[20] Wang F, Bornstein M, Hung K, Fan S, Chen X, Huang W (2018). Application of real-time surgical navigation for zygomatic implant insertion in patients with severely atrophic maxilla. J Oral Maxillofac Surg.

[21] Tahmaseb A, Wismeijer D, Coucke W, Derksen W (2014). Computer technology applications in surgical implant dentistry: a systematic review. Int J Oral Maxillofac Implants.

[22] Walker-Finch K, Ucer C (2020). Five-year survival rates for implants placed using digitally-designed static surgical guides: a systematic review. Br J Oral Maxillofac Surg.

[23] Schneider D, Sancho-Puchades M, Mir-Marí J, Mühlemann S, Jung R, Hämmerle (2019). A randomized controlled clinical trial comparing conventional and computer-assisted implant planning and placement in partially edentulous patients. Part 4: accuracy of implant placement. Int J Periodontics Restorative Dent.

[24] Schiroli G, Angiero F, Zangerl A, Benedicenti S, Ferrante F, Widmann G (2015). Accuracy of a flapless protocol for computer-guided zygomatic implant placement in human cadavers: expectations and reality. Int J Med Robot Comput Assist Surg.

[25] Corvello PC, Montagner A, Batista FC, Smidt R, Shinkai RS (2011). Length of the drilling holes of zygomatic implants inserted with the standard technique or a revised method: a comparative study in dry skulls. J Cranio-Maxillofac Surg.

[26] Van Camp P, Vrielinck L, Gemels B, Politis C (2018). Intraorbital haemorrhage following a secondary intervention at integrated zygomatic implants: a case report. Int J Surg Case Rep.

[27] Garcia Garcia B, Ruiz Masera J, Zafra Camacho F. Bilateral cutaneous fistula after the placement of zygomatic implants. Int J Oral Maxillofac Implants. 2016:e11–4. 10.11607/jomi.4202. 10.11607/jomi.4202 26478973

[28] Neugarten J, Tuminelli F, Walter L (2017). Two bilateral zygomatic implants placed and immediately loaded: a retrospective chart review with up-to 54-month follow-up. Int J Oral Maxillofac Implants.

[29] Smitkarn P, Subbalekha K, Mattheos N, Pimkhaokham A (2019). The accuracy of single-tooth implants placed using fully digital-guided surgery and freehand implant surgery. J Clin Periodontol.

